# Evaluation of Healthy Eating Index and Children's Diet Inflammatory Index according to asthma severity group

**DOI:** 10.1186/s12887-023-04507-y

**Published:** 2024-02-17

**Authors:** Nevra Koç, Nursena Ersoy, Hülya Yardimci, İlknur Külhaş Çelik, Ersoy Civelek

**Affiliations:** 1 Department of Nutrition and Dietetics, Gülhane Health Sciences of Faculty, Sağlık Bilimleri University, İstanbul, Turkey; 2 Ankara University, Institute of Health Sciences, Ankara, Turkey; 3 Department of Nutrition and Dietetics, Faculty of Health Sciences, Ankara University, Ankara, Turkey; 4 Division of Pediatric Allergy Immunology, Faculty of Medicine, Selcuk University, Konya, Turkey; 5 Division of Pediatric Allergy Immunology, Sağlık Bilimleri University, Ankara City Hospital, Ankara, Turkey

**Keywords:** Asthma, Asthma severity, Healthy eating index, Children’s dietary inflammatory index, Nutrients

## Abstract

**Background:**

Diet may contribute to better asthma control in children by impacting the immune and inflammatory pathophysiology. Therefore, this study aimed to investigate differences in nutrient intake, Children’s Dietary Inflammatory Index (C-DII), and dietary quality according to asthma severity.

**Materials and methods:**

Asthma severity, dietary inflammatory status, and diet quality were assessed in a sample of 202 children with asthma (55.6% males, aged 5–18 years) attending a pediatric allergy outpatient clinic. Asthma severity was evaluated according to the Global Initiative for Asthma criteria and categorized as mild, moderate, or severe. The Children’s Dietary Inflammatory Index (C-DII) and Healthy Eating Index (HEI-2010) were calculated based on information collected by the 24-h dietary recall method. Dietary quality was categorized as poor, moderate, or good diet according to HEI-2010.

**Results:**

The mean age of the participants was 9.6 ± 3.2 years. Children with severe asthma were younger on average (*p* < 0.05). Children with mild asthma had significantly higher fiber and iron intake than those with moderate asthma (*p* < 0.05). C-DII values did not differ significantly according to asthma severity (*p* > 0.05). Dietary quality was evaluated as moderate in 89.1% of the participants and also showed no difference based on asthma severity.

**Conclusions:**

These findings suggest that inflammatory status and diet quality may not affect asthma severity in children, highlighting the influence of various genetic and environmental factors on the association between diet and asthma severity. More comprehensive and longitudinal studies are needed to investigate the mechanisms linking diet and asthma.

## Introduction

Asthma is a common chronic disease in children [1]. In the International Study of Asthma and Allergies in Childhood (ISAAC) III, the global frequency of wheezing in the last 12 months was found to be 11.8% in the 6–7 years age group and 13.8% in the 13–14 age group [2]. Among children in Turkey, the lifetime prevalence of wheezing has been reported as 10.2–31.4%, the prevalence of wheezing in the last year as 3.1–15.8%, and the prevalence of physician-diagnosed asthma as 0.7–21.2% [3]. Factors such as low socioeconomic status, various pollutants, low awareness, and underdiagnosis may lead to differences in the prevalence of asthma. However, asthma management is recognized as a global public health problem [4].

The recent changes in dietary patterns worldwide may contribute to the increasing prevalence of asthma through systemic inflammation [5]. Although studies on particular foods and food groups are conducted in nutrition research, the effect of consuming foods in combination has also attracted attention [6]. As a result, the link between food quality and asthma is significant because it reflects the interaction of nutrients, their bioavailability, and the overall diet. Other than diet quality, there could be a number of confounding factors in this association [7]. For instance, Li et al. (2017) showed that better diet quality in adults, regardless of BMI, reduced asthma symptoms in never-smokers [8]. Similarly, Baptista Menezes et al. (2022) determined that diet quality had a greater impact on asthma symptoms than other individual components [9]. The European Academy of Allergy and Clinical Immunology (EAACI) stated that diet quality in childhood is associated with a decrease in asthma symptoms [10]. However, studies on this topic are limited.

The Mediterranean diet shows a protective effect against asthma risk or asthma symptoms, whereas the Western-style diet, characterized by low dietary quality, increases the risk of asthma [11, 12]. The Western-style diet is high in fat and low in fiber, vitamins/minerals, and antioxidants [13, 14]. Low antioxidant content reduces response capacity to oxidative stress; a high-fat diet can lead to respiratory hyperreactivity through proinflammatory cytokines; and a low-fiber diet can worsen allergic airway inflammation [15]. In addition, the Dietary Inflammatory Index (DII), developed to determine the inflammatory potential of the diet, has been associated with increased systemic inflammation and poorer lung function [16]. An antioxidant-rich diet with high dietary quality in asthma patients may reduce the severity of asthma through its anti-inflammatory potential [17].

When designing this study, we hypothesized that DII would be higher and dietary quality lower in pediatric patients with more severe asthma. The objectives of this study were to determine the nutrient intake, Children’s Dietary Inflammatory Index (C-DII) values, and dietary quality of children with asthma and evaluate differences in these variables according to asthma severity.

## Methods

### Study population and sample selection

This study was conducted with 202 participants aged 5–18 years who presented to the pediatric allergy outpatient clinic of the Ministry of Health Ankara Children’s Hematology and Oncology Training and Research Hospital. Before starting the study, ethical approval was obtained from the Ethics Committee for Non-Invasive Scientific Research (no: 2017/50). All study procedures were carried out September 2017 and November 2018 in accordance with the Declaration of Helsinki.. Patients with asthma who were diagnosed at least a year ago, used inhalers, were followed up on in this hospital, and were able to respond to the questionnaire personally or obtain a response from their companions were included in the study. Exclusion criteria were being diagnosed with asthma less than 1 year ago, being younger than 5 or older than 18, and inability to communicate with the patient or a parent/guardian. All children in the study participated voluntarily and met the inclusion criteria. Informed consent was obtained from all subjects and their parent/legal guardian(s).

### Study design

The study data were collected from the patients and parents using a paper-based questionnaire completed by face-to-face interview under the supervision of a dietician and doctor. The questionnaire consisted of sociodemographic characteristics, anthropometric measurements (weight and height), and one-day dietary intake record based on the 24-h recall method. The following sociodemographic information about the children was obtained: date and mode of birth, birth weight, duration of exclusive breastfeeding, and total duration of breastfeeding. Dietary intake, children’s Healthy Eating Index (HEI-2010) and C-DII values were calculated based on the diet data collected by 24-h recall.

Asthma was diagnosed according to the Global Initiative for Asthma (GINA) criteria by a specialist in pediatric allergy and immunology. Asthma severity was classified according to medication requirement as mild (steps 1 and 2), moderate (steps 3–4), or severe (step 5) as recommended in the current GINA guideline [18].

### Anthropometric measurements

Height (cm) and body weight (kg) were measured and recorded in an anthropometric data form. Anthropometric measurements were obtained from all participants in the morning on an empty stomach, without shoes and outer garments. If the children did not have an empty stomach on the day of study recruitment, they were invited back on another day for measurement. Weight was measured using SECA brand scale. Height was measured with a portable stadiometer while the children were standing against a wall, feet flat, and the head held with the Frankfort horizontal plane (line between inferior orbital margin and supratragal notch) parallel to the ground. Body mass index (BMI) percentiles for age and sex were calculated from the children’s weight and height data using the World Health Organization’s Anthroplus program [19]. BMI values below the 15th percentile were interpreted as underweight, between the 15th and 85th percentiles as normal, between the 85th and 97th percentiles as overweight, and above the 97th percentile as obese [20].

### Assessment of dietary intake

One-day intake records were obtained using the 24-h dietary recall method. The day of recording was taken on the days that families stated that this was the day their children typically consumed food. The researcher conducting the interview asked for detailed information about the foods consumed at meals and their amounts, using a food photograph catalog. Food amounts were quantified as number and units of measure, and the corresponding nutrient content per portion was determined using this data and a standard recipe book. The average energy and macro/micronutrient content in the food consumed was calculated using the Turkish Nutrition Information System (BeBiS) version 7.0.

### Healthy Eating Index (HEI-2010)

Dietary quality was assessed using the HEI-2010 based on the children’s 24-h food intake records. The HEI-2010 includes a total of 12 groups. The first 9 groups (total fruit, whole fruit, total vegetables, greens and beans, whole grains, dairy, total protein foods, seafood and plant proteins, fatty acids) assess dietary adequacy and the last 3 groups (refined grains, sodium, and empty calories [i.e., energy from solid fats, alcohol, and added sugars]) assess foods that should be consumed in moderation. Each of the adequacy groups has its own standard and scores increase with greater consumption. In the moderate intake groups, lower consumption increases the score. The HEI-2010 is evaluated out of 100 points. Dietary quality is classified as poor if the total HEI-2010 score is below 51, moderate if between 51–80, and good if above 80 [21].

### Children’s diet inflammatory index

The C-DII has been validated in pediatric populations [22]. C-DII values were calculated according to 1-day food intake records obtained using the 24-h recall method. The calculation has 25 nutrient parameters, including vitamin A, thiamine, riboflavin, niacin, vitamin B_6_, folic acid, vitamin B_12_, vitamin D, vitamin C, vitamin E, beta carotene, energy, carbohydrates, fiber, total fat, saturated fat, monounsaturated fats, polyunsaturated fats, cholesterol, protein, alcohol, iron (Fe), magnesium (Mg), selenium (Se), and zinc (Zn). However, alcohol was not included in the calculation as none of the children in the study sample consumed alcohol. Z-scores were determined by subtracting the standard mean of the children’s consumption from each child’s reported consumption and dividing this value by the global standard deviation. These z-scores were then converted to a scale of -1 (maximal anti-inflammatory) to + 1 (maximal pro-inflammatory) to minimize the right-leaning effect. Finally, this score was multiplied by the nutrient parameter effect score. The overall C-DII score for each child was determined by summing the C-DII scores specific to all of the nutritional parameters. Higher C-DII indicates higher proinflammatory status.

### Statistical analyses

The data were analyzed using IBM SPSS Statistics version 25 package software. Normally distributed data were given as mean and standard deviation, and non-normally distributed data were given as median and range (minimum—maximum values). Nominal data were expressed as number and percentage. In the analysis of continuous variables, ANOVA was used to compare means between more than two parametric groups and the Kruskal–Wallis test was used to compare median values between more than two non-parametric groups. Categorical variables were analyzed using the chi-square test. If the variables were found to be significant in these tests, a post-hoc comparison was performed using Tukey HSD and Bonferroni-corrected Dunn test, respectively.

## Results

The sociodemographic and clinical characteristics of the participants are shown in Table 1. Of the participants, 55.6% were male and the mean age was 9.6 ± 3.2 years. Mode of delivery was vaginal for 53.0% of the participants, and 79.2% had normal birth weight. The mean total duration of breastfeeding was 16.2 ± 10.9 months and the duration of exclusive breastfeeding was 5.3 ± 2.5 months. According to their current weight, 55.9% of the participants were at a normal weight and 37.7% were overweight or obese.

**Table 1:** Patient demographic and clinical characteristics

Characteristic	n (%)
**Sex**	
Female	91 (44.4)
Male	114 (55.6)
**Age (years),** mean ± *SD*	9.6 ± 3.2
5–6	36 (17.8)
7–10	93 (46.0)
11–14	51 (25.2)
15–17	22 (11.0)
**Mode of delivery**	
Vaginal	107 (53.0)
Cesarean	95 (47.0)
**Birth weight (g),** mean ± *SD*	3179.77 ± 645.47
Small for gestational age (< 2500 g)	9 (14.4)
Normal birth weight (2500–4000 g)	160 (79.2)
Large for gestational age (> 4000 g)	13 (6.4)
**Current weight (kg),** mean ± *SD*	36.5 ± 15.2
Underweight (< 15th percentile)	13 (6.4)
Normal (15th-85th percentile)	113(55.9)
Overweight (85th-97th percentile)	50 (24.8)
Obese (> 97th percentile)	26 (12.9)
**Exclusive breastfeeding time (months),** mean ± *SD*	5.3 ± 2.5
**Total breastfeeding time (months),** mean ± *SD*	16.2 ± 10.9

The comparison of the participants’ nutrient intake according to asthma severity is shown in Table 2. Children with mild asthma had significantly higher fiber and iron intake than those with moderate asthma (*p* < 0.05).

**Table 2:** Nutrient intake according to asthma severity

	**Mild (*n* = 58)**	**Moderate (*n* = 89)**	**Severe (*n* = 55)**	**p**
Energy (kcal/d)	1684.4 (736.7–4069.3)	1501.5 (703.5–4543.7)	1559.9 (736.6–3532.6)	0.399
Carbohydrate (g/d)	205.8 (83.0–627.8)	180.9 (65.4–844.7)	195.6 (66.0–529.1)	0.089
Protein (g/d)	56.6 (24.2–108.5)	50.9 (15.2–124.4)	47.5 (20.0–120.3)	0.320
Total fat (g/d)	63.0 (13.7–134.4)	60.7 (18.0–146.6)	58.7 (23.8–153.1)	0.959
Saturated fat (g/d)	20.3 (3.1–61.4)	22.7 (5.7–53.8)	22.6 (8.8–52.5)	0.450
Monounsaturated fatty acids (g/d)	19.6 (3.7–46.9)	20.0 (4.2–58.2)	19.9 (4.6–43.9)	0.859
Polyunsaturated fatty acids (g/d)	17.6 (2.1–49.2)	14.6 (2.1–55.5)	14.0 (2.7–49.2)	0.473
Cholesterol (mg/d)	228.3 (14.8–634.7)	184.3 (14.0–718.2)	155.5 (10.7–972.0)	0.230
Fiber (g/d)	18.0 (5.0–36.4)^a^	14.0 (2.8–64.0)^a^	16.4 (4.8–123.1)	0.032*
Thiamine (mg/d)	0.7 (0.3–1.5)	0.6 (0.2–2.1)	0.6 (0,3–2.2)	0.056
Riboflavin (mg/d)	1.1 (0.3–4.3)	1.1 (0.3–2.7)	1.0 (0.5–3.2)	0.630
Niacin (mg/d)	7.2 (2.2–40.4)	6.2 (1.7–33.9)	6.9 (1.8–32.5)	0.159
Pyridoxin (mg/d)	1.1 (0.4–7.1)	0.9 (0.2–9.0)	0.9 (0.4–2.1)	0.093
Cobalamin (mg/d)	2.2 (0.0–62,5)	2.5 (0.0–9.0)	2.4 (0.4–8.9)	0.650
Vitamin A (µg/d)	555.3 (93.2–16883.9)	572.2 (49.2–3408.8)	511.9 (77.4–4116.5)	0.679
Vitamin C (mg/d)	69.8 (1.1–337.0)	72.7 (0.7–270.3)	76.2 (0.7–341.5)	0.971
Vitamin E (mg/d)	13.9 (2.2–38.2)	13.3 (1.7–54.4)	11.9 (2.9–46.6)	0.097
Iron (mg/d)	8.2 (4.0–19.4)^b^	7.3 (2.8–21.8)^b^	7.4 (3.6–59.2)	0.019*
Magnesium (mg/d)	207.6 (80.8–461.3)	180.9 (53.1–427.3)	188.6 (83.2–1005.0)	0.228
Zinc (mg/d)	7.3 (3.8–17.3)	6.7 (2.6–16.7)	6.7 (3.1–15.2)	0.591

^*^Matching letters indicate significant difference between groups. Tukey HSD and Bonferroni-corrected Dunn tests were used for post-hoc analyses

Comparisons of the children’s demographic information, dietary quality, and diet inflammatory status according to asthma severity are shown in Table 3. Children with mild asthma severity were older on average than those with severe asthma severity (*p* < 0.05). Children with severe asthma had longer total breastfeeding duration compared to those with moderate asthma (*p* < 0.05). Furthermore, when forced expiratory volume in the first second (FEV_1_) was classified as low (< 70% of predicted) and high (> 70% of predicted), there was no statistically significant difference between children in the low and high FEV_1_ groups in terms of HEI-2010 (*p* = 0.409) or C-DII (*p* = 0.141).

**Table 3:** Demographic features, dietary quality and inflammatory status according to asthma severity

	**Mild (*n* = 58)**	**Moderate (*n* = 89)**	**Severe (*n* = 55)**	**p**
**Age (years),** mean ± SD	10.5 ± 3.3^a^	9.4 ± 3.0	8.8 ± 3.3^a^	0.012*
**Total breastfeeding time (months),** median (range)	12.0 (0.0–30.0)	12.0 (0.0–36.0)^b^	24.0 (0.0–36.0)^b^	0.013*
**BMI,** n (%)				
Underweight	4 (7.0)	8 (8.9)	3 (5.5)	0.884
Normal	32 (56.1)	53 (58.9)	28 (50.9)	
Overweight	14 (24.6)	19 (21.1)	17 (30.9)	
Obese	7 (12.3)	10 (11.1)	7 (12.7)	
**HEI-2010 score,** median (range)	57.4 (45.2–74.8)	57.1 (45.9–73.1)	57.2 (49.1–71.4)	0.887
**HEI-2010 classification,** n (%)				
Poor	7 (12.3)	10 (11.2)	5 (9.1)	0.607
Moderate	50 (87.7)	79 (88.8)	50 (90.9)	
Good	-	-	-	
**C-DII,** mean ± *SD*	1.3 ± 0.7	1.2 ± 0.7	1.4 ± 0.8	0.435

*BMI* Body Mass Index, *C-DII* Children’s Dietary Inflammatory Index, *HEI* Healthy Eating Index

^*^Matching letters indicate significant difference between groups. Tukey HSD and Bonferroni-corrected Dunn tests were used for post-hoc analyses

## Discussion

In this study, although C-DII values were higher in children with severe asthma compared to those with mild asthma, dietary inflammatory status and quality did not differ according to asthma severity. The majority of children with asthma had moderate dietary quality.

Asthma severity is an important indicator for evaluating asthma care and management [23].The decrease in asthma severity with age observed in our study may have related to the high frequency of infections, a known trigger of asthma, in young children. In addition, we noted a longer total breastfeeding duration among children with severe asthma. Breast milk is rich in bioactive components such as oligosaccharides, cytokines, enzymes, and immune cells. This could impact the maturation of the infant’s gut microbiota and immune system, which may be involved in the development of asthma [24]. However, these bioactive factors are affected by factors such as ethnicity, diet, body composition, smoking, medical history, and geographical location [25]. Furthermore, a variety of factors such as drug use, passive smoking, disease, and stress have an impact on the gut flora [26]. These differences may explain the higher total breastfeeding time in children with severe asthma compared to those with mild asthma. In addition, the possible introduction of complementary foods later in children with high asthma severity may have contributed, though this was not questioned in our study.

Children who are obese have a higher risk of asthma. Obesity may reduce respiratory capacity, leading to shortness of breath and respiratory tract hypersensitivity [27]. In addition, higher BMI is associated with increased leptin levels. Leptin may aid in the prediction of asthma severity [28]. However, in this study, there was no difference in children’s BMI category according to level of asthma severity. Adipose tissue, which increases with body weight, can affect inflammatory processes. However, inflammatory processes are influenced by various hormones and systems. The lack of difference in this study may be due to the complexity of the inflammatory process and the fact that the majority of the participants were pubescent adolescents with normal weight.

Dietary patterns have important impacts on metabolic processes and immune health through the gut microbiota [29]. It has been previously reported that adherence to the Mediterranean diet and longer breastfeeding time reduce the incidence of asthma in children [30]. Another study showed that children with asthma had fewer attacks and associated complications after adherence to the Mediterranean diet [31]. Similarly, various studies stated that adherence to the Mediterranean diet was negatively associated with asthma symptoms in children [32, 33]. In addition, children who consume refined and processed foods were found to have higher levels of inflammatory markers compared to children who eat an abundance of fruits and vegetables [34, 35].

Limited consumption of refined flour and sugar in the Mediterranean diet lowers the glycemic index. It prevents hyperinsulinemia, which promotes the formation of pro-inflammatory eicosanoids [36]. In addition, decreased consumption of unhealthy fats limits inflammation, while omega-3 and omega-6 fatty acids reduce inflammatory cytokines and supports the differentiation of T helper cells [37]. The Mediterranean diet is also rich in vitamins, minerals, fiber, and antioxidants. Thus, it supports the immune response, blocks the bronchial inflammatory response, and contributes to the symbiotic gut flora, thereby inducing oral tolerance. These features of the Mediterranean diet, which has high dietary quality, may be associated with asthma severity [38]. However, in contrast to these results, there there are no differences in diet quality between categories of asthma severity in the present study. The disparities in our study’s findings could be attributed to children’s typically poor dietary quality.

Only the children’s fiber and iron intake differed according to asthma severity (*p* < 0.05). Fiber may support immune health through metabolic modulation of gut microbiota [10]. This may explain the lower intake of fiber in children with moderate asthma compared to children with mild asthma. Dietary iron, which has pro-inflammatory activities, is a significant component of the dietary inflammatory score [39]. However, iron intake was higher in the mild asthma group in this study. This emphasizes the importance of the inflammatory status of the diet as a whole, despite the inflammatory qualities of certain foods. In addition, the majority of children were found to have moderate dietary quality.. These results show that children’s eating habits do not meet nutritional requirements. Determining causality in follow-up studies evaluating asthma severity and dietary quality may yield more accurate results. Parents in particular can be educated on the characteristics of the Mediterranean diet and the health benefits it will provide.

When dietary inflammatory status and asthma severity were examined, no significant differences were detected between the groups. It has been shown that asthma symptoms increase with greater inflammatory potential of the diet in children aged 5 to 14 years [40]. A higher DII score, which is an indicator of a proinflammatory diet, may be associated with more severe symptoms due to increased systemic inflammation [16]. However, C-DII scores of the children in our study did not differ according to asthma severity. This may be due to the generally high dietary inflammatory potential and predominantly moderate asthma among the children sampled.

## Limitations

Although this study provides important data on the relationship between asthma severity and dietary quality and inflammatory status, there are several limitations. Firstly, because of the cross-sectional design, the results may not be generalizable to the general population. Secondly, both HEI and C-DII may be affected by seasonality. Additionally, since the 24-h recall method was used, short-term food intake was focused on. However, taking it on days when it is similar to their daily intake is a strength of the study. In addition, determining the children’s time of diagnosis and whether have a family history of asthma may yield more definitive results. Finally, studies conducted in larger population groups are needed to confirm our findings. Despite these limitations, the study brings new perspectives to the current literature.

## Conclusion

Nutrition may contribute to better asthma control in children by affecting the immune and inflammatory pathophysiology. However, in the present study, diet quality and inflammatory status did not differ by asthma severity. This relationship remains unclear because of the lack of longitudinal studies and adequate dietary control.

## Data Availability

The datasets generated and/or analysed during the current study are not publicly available due to privacy or ethical restrictions, but are available from the corresponding author on reasonable request.
